# Immunosuppressive Glycodelin A is an independent marker for poor prognosis in endometrial cancer

**DOI:** 10.1186/1471-2407-13-616

**Published:** 2013-12-30

**Authors:** Miriam Lenhard, Sabine Heublein, Christiane Kunert-Keil, Thomas Vrekoussis, Isabel Lomba, Nina Ditsch, Doris Mayr, Klaus Friese, Udo Jeschke

**Affiliations:** 1 Department of Obstetrics and Gynecology, Ludwig-Maximilians-University Munich, Campus Grosshadern, Marchioninistr. 15, 81377 Munich, Germany; 2 Department of Obstetrics and Gynecology, Ludwig-Maximilians-University Munich, Campus Innenstadt, Maistr. 11, 80337 Munich, Germany; 3 Department of Orthodontics, Technische Universität Dresden, Fetscherstr. 74, 01309 Dresden, Germany; 4 Department of Pathology, Ludwig-Maximilians-University Munich, Thalkirchner Str. 36, 80337 Munich, Germany

**Keywords:** Endometrial cancer, Glycodelin, Glycodelin A, Immunohistochemistry, In situ hybridization, Prognosis

## Abstract

**Background:**

Knowledge on immunosuppressive factors in the pathogenesis of endometrial cancer is scarce. The aim of this study was to assess Glycodelin (Gd) and its immunosuppressive isoform Glycodelin A (GdA) in endometrial cancer tissue and to analyze its impact on clinical and pathological features and patient outcome.

**Methods:**

292 patients diagnosed and treated for endometrial cancer were included. Patient characteristics, histology and follow-up data were available. Gd and GdA was determined by immunohistochemistry and in situ hybridization was performed for Gd mRNA.

**Results:**

Endometrial cancer shows intermediate (52.2%) or high (20.6%) expression for Gd in 72.8%, and GdA in 71.6% (intermediate 62.6%, high 9.0%) of all cases. The glycosylation dependent staining of GdA is tumour specific and correlates with the peptide-specific Gd staining though neither of the two is associated with estrogen receptor, progesterone receptor or clinic-pathological features. Also Gd protein positively correlates with Gd mRNA as quantified by in situ hybridization. Gd positive cases have a favourable prognosis (p = 0.039), while GdA positive patients have a poor outcome (p = 0.003). Cox-regression analysis proofed GdA to be an independent prognostic marker for patient survival (p = 0.002), besides tumour stage, grade and the concomitant diagnosis of hypertension.

**Conclusion:**

Gd and GdA are commonly expressed in endometrial cancer tissue and seem to be of relevance in tumourigenesis. They differ not only in glycosylation but also in their biological activity, since only GdA holds prognostic significance for a poor overall survival in endometrial cancer patients. This finding might be explained by GdAs immunosuppressive capacity.

## Background

Endometrial cancer is the fourth common carcinoma in women following cancer of breast, colon and lung and accounts for 5.6% of all malignancies [1]. The diagnosis of endometrial cancer is typically made at postmenopausal age [2] and its 5-year survival ranges between 75 and 83% [2].

Some risk factors for the development of endometrial cancer have been described [3-8], though the exact mechanisms in tumourigenesis are by far not explained. A fast tumour progression is most likely favoured by local immunosuppression, which decreases the body’s own anti-tumour immunoreactivity. Until today little is known about tumour induced, local immunosuppression in endometrial cancer.

Glycodelin (Gd), also known progestagen-associated endometrial protein, is a glycoprotein with immunosuppressive capacity, which is mainly produced in reproductive tissue [9,10]. Four different isoforms have been described: GdS (in seminal vesicles and seminal plasma) [11], GdA (in endometrium/decidua, amniotic fluid, maternal serum) [12,13], GdF (in follicular fluid und oviduct) [14] und GdC (in the cumulus oophorus) [15]. The isoforms share a common protein backbone but differ in glycosylation and biological activity [16,17].

GdA holds several immunosuppressive abilities, which are best characterized in reproductive medicine [18]. These include the suppression of lymphocyte proliferation and inhibition of T- and B-cell activity [19-21]. Moreover, the induction of apoptosis via GdA has been investigated [22].

Recently, we found GdA to be of prognostic significance in ovarian cancer [23]. So far, there are very few results on endometrial cancer cells and Gd or GdA [24] and no clinical data on endometrial cancer. Therefore, the aim of this study was to assess the expression of Gd on mRNA and protein level. Further, we aimed to specify the proportion of the immunosuppressive glyko-modification GdA in tissue samples of a large cohort of endometrial cancer patients by using an extensively validated anti GdA antibody. Finally, we aimed to analyse the impact of Gd/GdA positivity on clinical and pathological features including patient outcome.

## Methods

### Patients

Formalin fixed paraffin embedded (FFPE) tissue of 292 endometrial cancer patients (Table 1) was available. Most patients presented with early stage disease at primary diagnosis (Table 1). 72.6% of patients (n = 212) showed a Type I carcinoma with endometrioid histology. Among the remainder there were 7.9% with serous, 4.1% with mucinous, 1.7% with clear cell histology and 0.3% with squamous cell histology. 11.6% were classified as mixed and 1.7% as undifferentiated carcinomas. Patients were also evaluated for concomitant diseases and presented with hypertension in 39.7%, obesity in 30.5% and diabetes in 11.3% of all patients.

**Table 1:** **Patient characteristics: Immunohistochemical staining for oestrogen receptors (ER) (ER alpha, ER beta) and progesterone receptors (PR) (PR-A and PR-B) were performed and analysed as previously published by our research group**[25]

**Grade (%)**	**1**	**147 (51.2)**
(n = 287)	**2**	93 (32.4)
	**3**	47 (16.4)
**FIGO stage (%)**	**I**	219 (75.0)
(n = 292)	**II**	21 (7.2)
	**III**	44 (15.1)
	**IV**	8 (2.7)
**Histology (%)**	**Endometrioid**	212 (72.6)
(n = 292)	**Serous**	23 (7.9)
	**Clear cell**	5 (1.7)
	**Mucinous**	12 (4.1)
	**Squamous cell**	1 (0.3)
	**Mixed**	34 (11.6)
	**Undifferentiated**	5 (1.7)
**Patient age ± sem [y] (range)**		65.1 ± 0.6 (35.6-88.1)
**Deaths (%)**		160 (54.8)
**Survival ± sem [y] (95% CI)**		13.6 ± 0.5 (12.6-14.6)
**Follow up ± sem [y] (95% CI)**		13.8 ± 0.3 (13.1-14.5)
**Glycodelin (%)** (n = 291)	**Low**	79 (27.1)
	**Intermediate**	152 (52.2)
	**High**	60 (20.6)
**Glycodelin A (%)** (n = 289)	**Low**	82 (28.4)
	**Intermediate**	181 (62.6)
	**High**	26 (9.0)
**ER alpha (%)** (n = 292)	**Positive**	133 (45.5)
**ER beta (%)** (n = 292)	**Positive**	40 (13.7)
**PRA (%)** (n = 292)	**Positive**	121 (41.4)
**PRB (%)** (n = 292)	**Positive**	134 (45.9)
**Co-morbidities**	**Hypertension (%)**	116 (39.7)
	**Diabetes (%)**	33 (11.3)
	**Obesity (%)**	89 (30.5)
**Lymphangiosis (%)** (n = 292)	**Positive**	27 (9.2)
	**Negative**	263 (90.1)
	**Unknown**	2 (0.7)
**Hemangiosis (%)** (n = 292)	**Positive**	8 (2.7)
	**Negative**	281 (96.2)
	**Unknown**	3 (1.0)
**Radiotherapy (%)** (n = 292)	**Yes**	116 (39.7)
	**No**	170 (58.2)
	**Declined**	6 (2.1)
**Chemotherapy (%)** (n = 292)	**Yes**	7 (2.4)
	**No**	283 (96.9)
	**Declined**	2 (0.7)

### Assay methods

#### Immunohistochemistry

Immunohistochemical (IHC) staining has been described previously by us [23,26,27]. Glycosylation dependant staining differences were assessed using the polyclonal Gd and the monoclonal GdA antibody (A87-B/D2) [28]. Specificity of GdA binding was analyzed by Western blot analysis [29-31]. This antibody is suitable for the detection of GdA in endometrial tumour tissues [26]. Our former investigation showed that A87-B/D2 seems to be less restricted to GdA carbohydrate structures than other monoclonal antibodies made in our laboratories, although none of the three monoclonal antibodies recognize GdS or other pregnancy-related glycoproteins such as hCG or transferrin isolated from amniotic fluid [26].

Formalin fixed paraffin embedded tissue sections were dewaxed with xylol and endogenous peroxidase activity was quenched by dipping in 3% hydrogen peroxide (Merck, Darmstadt, Germany) in methanol for 20 min. Then sections were rehydrated in descending concentrations of alcohol. For GdA staining epitope retrieval was performed in a pressure cooker using sodium citrate buffer (5 min, pH 6.0). Following PBS washes samples were blocked as described in Table 2 and incubated with the primary antibodies (Table 2). Then samples were further processed as per manufacturer’s instructions. Finally, immunoreactivity was visualized using diaminobenzidine (Dako, Glostrup, Denmark), slides were counterstained using haematoxylin, dehydrated in ascending concentrations of alcohol, xylol treated and covered. Positive (placenta tissue) and negative (species matched pre-immune sera) controls were always included in the analysis (Additional file 1).

**Table 2:** **Immunohistochemistry: Antibodies detecting Gd or GdA were published by Jeschke et al. 2006**[26]**and Jeschke et al. 2005**[32]**, respectively**

**Antibody**	**Host/clonality**	**Epitope retrieval**	**Blocking**	**Dilution/incubation**	**Negative control**	**Reaction system**
Gd [32]	Rabbit/polyclonal	Not performed	Reagent 1 (5 min)	1: 800 (diluted in Dako antibody diluent)/o.n. (4°C)	Rabbit pre-immune serum (Dako)	Vectastain elite (rabbit IgG) kit (Burlingame, CA)
GdA [26]	Mouse/monoclonal	Citrate buffer (5 min, pressure cooker)	1.5% horse serum (20 min)	1:2000 (diluted in Dako antibody diluent)/o.n. (4°C)	Mouse pre-immune serum (Dako)	Vectastain elite (mouse IgG) kit (Burlingame, CA)

#### Preparation of riboprobes

Preparation of riboprobes was performed as described previously [27,33]. In short, a 227-bp fragment of the Gd cDNA (positions +41 to +268) was cloned into the EcoR1 restriction sites of pBluescript SK (Stratagene, Amsterdam, The Netherlands) and labelled with digoxigenin (DIG) by in vitro transcription using the DIG RNA labeling Kit (SP6/T7; Roche Biochemicals, Mannheim, Germany). The antisense cRNA probe binds in situ to Gd-mRNA and was utilized for Gd-mRNA detection. The sense cRNA probe was used as negative control.

#### In situ hybridization

Non-radioactive in situ-hybridization (ISH) analysis of Gd was performed on paraffin sections as described previously [27,28,34]. Briefly, paraffin sections were deparaffined, rehydrated and permeabilized by pepsin digestion (750 mg/ml pepsin in 0.2 M HCl, 37C, 30 min). Postfixation (paraformaldehyde 4%, 20 min, 4C) was followed by acetylation using 0.25% acetic anhydride in triethanolamine (0.1 M, pH 8.0, 15 min). After dehydration in an ascending series of alcohol, the sections were hybridized for 16 hr (56°C) in a solution containing 50% formamide, 50% solution D (4 M guanidine thiocyanate, 25 mM sodium citrate, pH 7.0), 0.5% blocking reagent, 210 mg/ml t-RNA derived from E. coli MRE 600, and 125 ng DIG-labeled cRNA probe. After washing with decreased concentrations of SSC (203 SSC: 3 M NaCl, 0.3 M sodium citrate, pH 7.4), sections were incubated 1 hr with blocking reagent (all from Roche Biochemicals).

Bound riboprobe was visualized by incubation with alkaline phosphatase-conjugated anti-DIG antibody (Roche Biochemicals) and subsequent substrate reaction using 5-bromo-4-chloro-3-indolyl phosphate/nitroblue-tetrazolium chloride [27,28,34].

### Specimen characteristics

All tissue samples (n = 292) were gained at surgery in patients who had been treated for primary endometrial cancer at our institution between 1990 and 2001. Histological evaluation including tumour staging and grading were performed by an experienced gynaecologic pathologist (D.M.) according to the criteria of the International Federation of Gynaecologists and Obstetricians (FIGO) and the World Health Organization (WHO).

### Study design

Tissue samples of endometrial cancer tissue gained at surgery at the Department of Obstetrics and Gynaecology of the Ludwig-Maximilians University Munich between 1990 and 2001 were randomly retrieved from the archive. FFPE material was stained for Gd, GdA or underwent ISH for Gd mRNA; clinical data were analysed retrospectively. Patients with uterine sarcoma were excluded from the study. Patient’s clinical data (Table 1) were available from patient charts, aftercare files and tumour registry database information. Mean follow-up time was 13.8 years (95% CI: 13.1-14.5) with 160 deaths. Mean overall survival was 13.6 years (95% CI: 12.6-14.6). The outcome assessed was patient survival.

The study has been approved by the ethics committee of the Ludwig-Maximilians University Munich (approval number: 063–13) and has been carried out in compliance with the guidelines of the Helsinki Declaration of 1975.

### Statistical analysis methods

Statistical analysis was performed using SPSS 20.0 (PASW Statistic, Ehningen, Germany). The non-parametric Kruskal-Wallis rank-sum test and for pairwise comparisons the non-parametric Mann–Whitney-U rank-sum test were used to test for differences between groups. Correlation analysis was performed using Spearman correlation. For the comparison of survival times, Kaplan-Meier curves were drawn. The chi-square statistic of the log-rank test was calculated to test differences between survival curves for significance. Multivariate analysis for prognostic value was performed using the Cox-regression model. Mean values are displayed ± standard error and p values below 0.05 were considered statistically significant.

Immunohistochemical staining was assessed using a semiquantitative immunoreactive score (IRS) according to Remmele and Stegener [35]. The IRS, ranging from 0 to 12, multiplicates staining intensity (graded as 0 = no, 1 = weak, 2 = moderate, and 3 = strong staining) and the percentage of positively stained cells (0 = no, 1 ≤ 10%, 2 = 11–50%, 3 = 51–80% and 4 ≥ 81% cells). The slides were reviewed in a blinded fashion by two independent observers. Intermediate positivity was set as median IRS ± one IRS unit, while low to negative immunoreactivity was assumed for IRS ≤ median IRS - two IRS units and high positivity was attributed for IRS ≥ median IRS + two IRS units (Additional file 2).

Gd mRNA expression was analysed automatically in a computer aided procedure as published previously by our group [27,28,34]. Briefly, five digital pictures were taken randomly from each tissue section (3CCD color camera, HV-C20M, Hitachi, Denshi, Japan, and Axiolab, Carl Zeiss, Jena, Germany). Optical density of white background colour was attuned to 250 to standardize measurements. Mean optical density and Gd positive pixels were determined by KSRun software (imaging system KS400, release 3.0, Zeiss). In accordance to the IRS system Gd positive pixels were ranked in nine groups representing lowest (group 1) to highest (group 9) Gd mRNA expression.

## Results

Endometrial cancer tissue of 292 patients (Table 1) was available. Data of 291 cases analysed for Gd, 289 cases stained for GdA and 254 cases analysed for Gd mRNA were included in the statistical analysis. Remaining cases (IHC: Gd: n = 1, GdA: n = 3 and ISH: Gd mRNA: n = 38) had to be excluded due to technical reasons. Mean patient age at primary diagnosis was 65.1 ± 0.6 years (range 35–88 years) and further patient characteristics are listed in Table 1.

### Gd mRNA expression in endometrial cancer tissue

**Figure 1:**
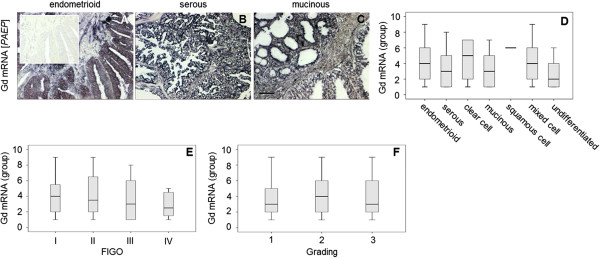
**Gd mRNA (PAEP) was detected in endometrial cancer tissue by in situ hybridization.** Representative microphotographs of Glycodelin (Gd) mRNA (*PAEP*, progestagen-associated endometrial protein) as detected by in situ hybridization in different histological subtypes **(A-C)** of endometrial cancer tissue are shown. Samples were treated with an antisense riboprobe recognizing Gd mRNA **(A-C)** or with the complementary sense riboprobe as a negative control (insert in A), respectively. Mean optical density of Gd mRNA signal has been quantified in a semi-automated manner and Gd positive pixels were determined by KSRun software. Gd mRNA positivity in dependence of histological subtype **(D)**, FIGO stage **(E)** and grading **(F)** is illustrated using box plot diagrams. Scale bar in C represents 100 μm and refers to **A-C**.

Intermediate (37.8%) or high (24.0%) expression of Gd mRNA (PAEP, progestagen-associated endometrial protein) was detected in the majority of cases investigated here (Figure 1). Though mRNA in situ hybridization revealed Gd transcripts to predominantly localize to the tumour epithelium, no significant difference in Gd mRNA expression was detected among histological tumour subtypes (Figure 1). In the current study positivity for Gd mRNA was neither statistically associated with histological tumour grade nor with the patients’ FIGO stage.

### Gd and GdA in endometrial cancer tissue

**Figure 2:**
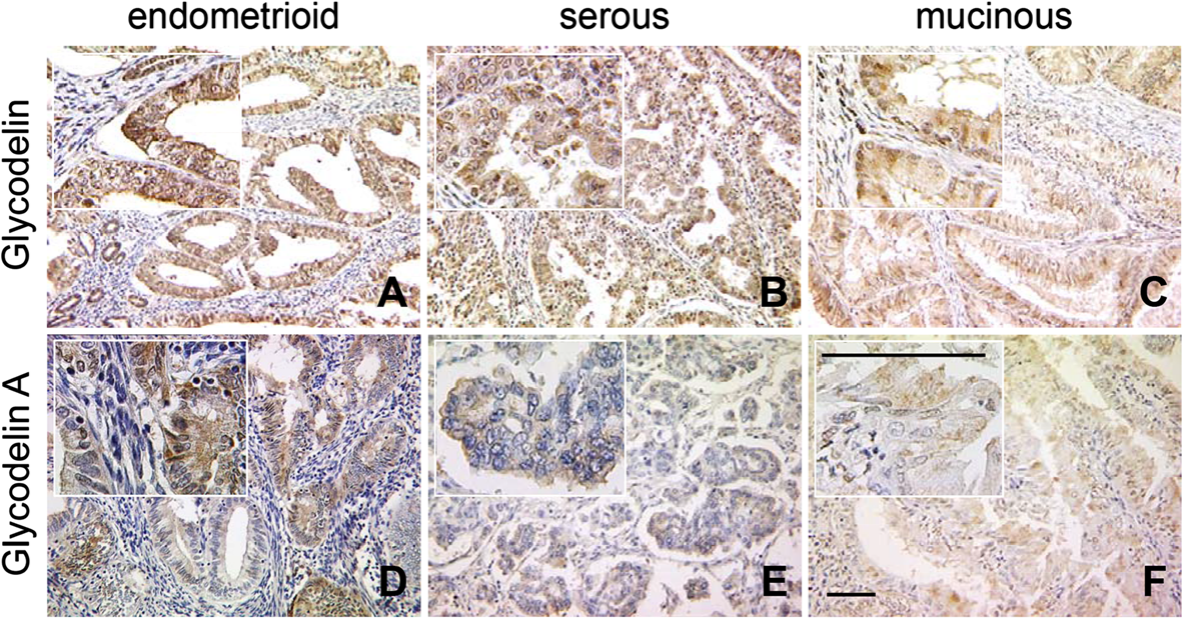
**Glycodelin and Glycodelin A protein was detected in endometrial cancer tissue by immunohistochemistry.** Representative microphotographs of Glycodelin (Gd, **A-C**) and its immunosuppressice glyco-variant Glycodelin A (GdA, **D-F**) as detected by immunohistochemistry in different histological subtypes of endometrial cancer tissue are shown. Pan-Glycodelin as well as its immunosuppressive glyco-variant Glycodelin A was found to be predominantly produced by epithelial components of endometrial carcinomas. Scale bars in **F** represent 100 μm and refer to **A-F**.

Using a polyclonal antiserum we also proofed presence of Gd protein in endometrial tissue. Immunohistochemical staining showed endometrial cancer tissue to be intermediately or highly positive for Gd in 52.2% or 20.6% (Figure 2, Table 1), respectively. A significant proportion of endometrioid carcinoma cases were observed to produce an immunosuppressive Gd glyocmodification, termed GdA. The latter was present at intermediate or high levels in 62.6% or 9.0% of cases, respectively. Inspite the fact that immunoreactivity for the Gd protein was positively correlated with Gd mRNA expression (Correlation coefficient 0.155, p = 0.013), no such association was observed when Gd mRNA was correlated with the glyco-variant GdA. However, GdA immunoreactivity was closely correlated with Gd protein expression (Correlation coefficient 0.249, p < 0.001).

**Figure 3:**
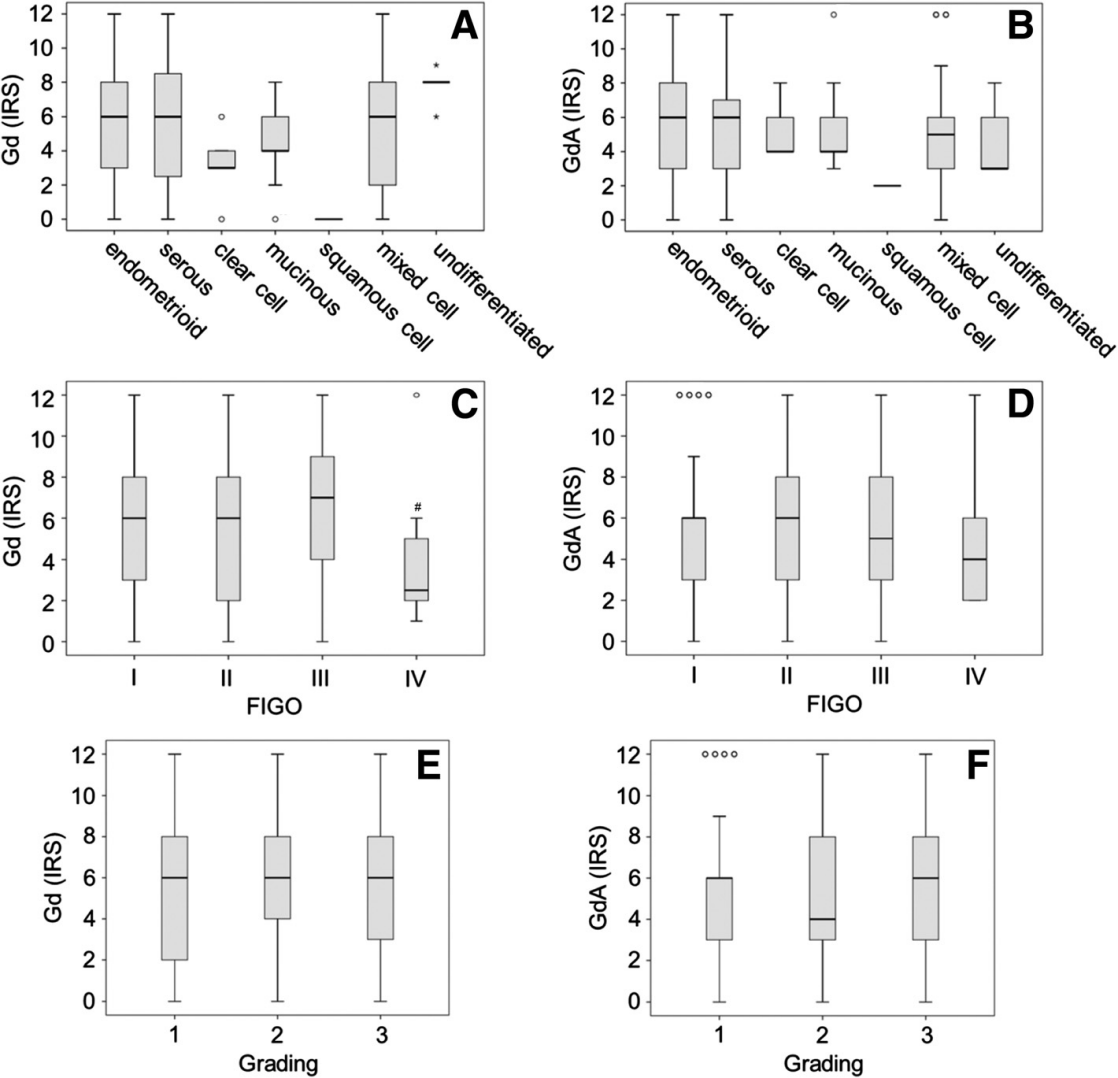
**Glycodelin as well as its immunosuppressive glyco-variant Glycodelin A protein was analysed and quantified in endometrial cancer tissue.** Quantification of Glycodelin (Gd; **A, C, E**) and its immunosuppressive glyco-variant Glycodelin **A** (GdA; **B, D, F**) by immunohistochemistry is shown. Gd/GdA was visualized in endometrial carcinoma tissue of different histological subtypes **(A, B)**, FIGO stages **(C, D)** or tumour grades **(E, F)**. Gd and GdA were detected by immuno-histochemistry and quantified employing an immunoreactive score (IRS) ranging from 0 (lowest) to 12 (highest). Significant differences (p < 0.05) as determined by Mann–Whitney Test are indicated by #.

The highest median Gd expression was noted for the undifferentiated histological subtype (median IRS 8.0; mean IRS 7.80 ± 0.49, Additional file 3), followed by the endometrioid (median IRS 6.0; mean IRS 5.9 ± 0.23), the serous (median IRS 6.0; mean IRS 5.74 ± 0.76) and the mixed cell type (median IRS 6.0; mean IRS 4.97 ± 0.60), though differences among Gd expression and the histological subtypes did not reach statistical significance (p > 0.05) (Figures 2 and 3). Comparable results were observed for GdA expression and the histological subtype (Figure 2 and 3). The most common histological subtypes (endometrioid and serous) show median GdA expression of IRS 6.0. Also, no statistically significant differences in GdA expression were observed among the different histological subtypes (p > 0.05) (Figures 2, 3 and Additional file 3).

Interestingly, there is a significant reduction in Gd expression observed from FIGO III to FIGO IV (p = 0.044) (Figure 3). However, overall Gd/GdA immunoreactivities comparing cases of low vs. high FIGO stage were not significantly different (Additional file 4). There were no significant differences in Gd and GdA expression between different tumour grades (Figure 3). Immunoreactivity of Gd or GdA staining was not significantly different comparing cases being negative vs. positive for ERs, PRs or co-morbidities.

### Prognostic value

**Figure 4:**
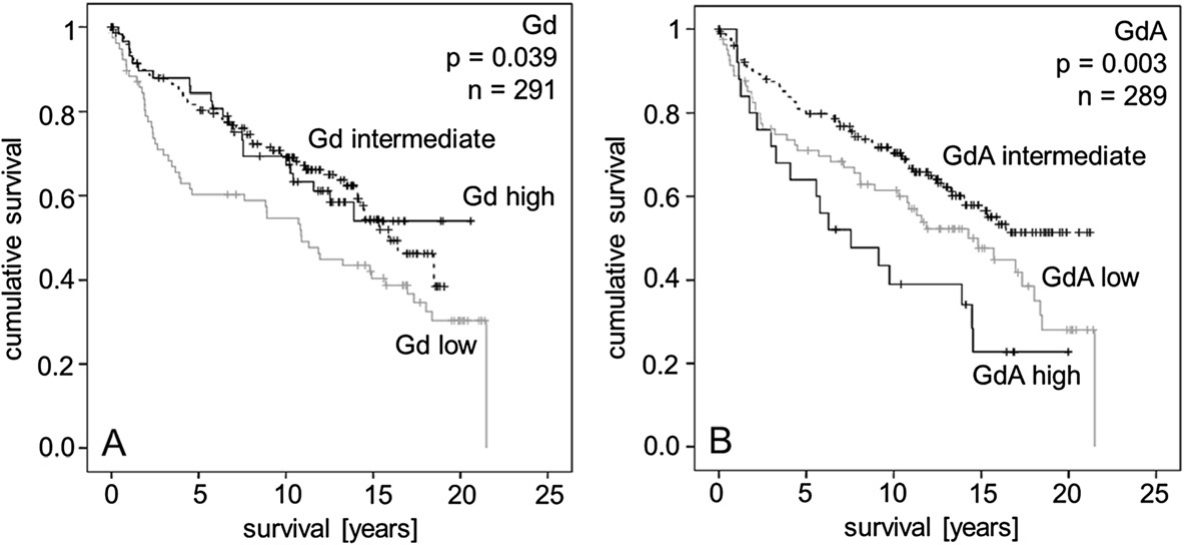
**Kaplan Meier survival analyses were performed for Glycodelin and Glycodelin A in endometrial cancer patients.** Overall survival of patients with low, intermediate and high Glycodelin A **(A)** and Glycodelin **(B)** protein expression as detected by immunohistochemistry is shown.

Statistical analysis was also performed to test for a prognostic value of Gd or GdA expression. Univariate Kaplan Meier analysis revealed a good prognosis for intermediate and high Gd expression (p = 0.039) (Figure 4A). In contrast, highly positive GdA endometrial cancer patients had a poor outcome compared to intermediate and low GdA expression (p = 0.003) (Figure 4B). Gd mRNA expression was not significantly associated with patients’ outcome.

Besides tumour stage, grade and the concomitant diagnosis of hypertension (each p < 0.05), Cox-regression analysis (Table 3) showed GdA to be an independent prognostic marker for patient survival (p = 0.002, 95% CI 1.362-3.943).

**Table 3:** Multivariate COX regression analysis: Patient survival was analysed by multivariate COX regression analysis

			**95% CI**		
**Covariate**	**Coefficient (b**_**i**_**)**	**[HR Exp(b**_**i**_**)]**	**Lower**	**Upper**	**P-value**
**FIGO stage**					**<0.001**
I	(0.000)	(1.00)			
II	−3.064	.047	.011	.192	**<0.001**
III	−3.025	.049	.010	.233	**<0.001**
IV	−1.810	.164	.040	.669	**.012**
**WHO grade**					**.023**
1	(0.000)	(1.00)			
2	−.820	.440	.246	.790	**.006**
3	−.561	.571	.325	1.002	.051
**Histology**					0.068
Endometrioid	(0.000)	(1.00)			
Serous	−.297	.743	.233	2.372	.616
Clear cell	−.069	.934	.260	3.349	.916
Mucinous	−1.423	.241	.024	2.403	.225
Squamous cell	.074	1.077	.260	4.470	.918
Mixed cell	3.167	23.742	2.245	251.079	**.008**
Undifferentiated	−.163	.850	.255	2.828	.790
**Lymph node** metastasis	−.732	.481	.209	1.109	.086
**Age** (≤50 y vs. >50 y)	1.939	6.953	.909	53.204	.062
**Diabetes**	.451	1.570	.880	2.800	.127
**Obesity**	−.065	.937	.600	1.462	.774
**Hypertension**	.454	1.575	1.043	2.380	**.031**
**Lymphangiosis**	.216	1.241	.631	2.443	.532
**Hemangiosis**	.681	1.975	.476	8.187	.348
**ER alpha**	−.031	.970	.652	1.442	.880
**PRA**	−.156	.855	.572	1.279	.446
**GdA**	.840	2.317	1.362	3.943	**.002**
**Gd**	−.298	.743	.456	1.209	.232

Significant results are shown in bold.

## Discussion

Endometrial cancer can be subdivided into two histological subtypes, the estrogen-associated Type I and the estrogen-independent Type II carcinoma [36,37]. The most common cause for endometrial Type I carcinoma is thought to be an excess of estrogens, which are inadequately antagonized by gestagens [38]. Therefore obesity, polycystic ovarian syndrome, menopausal hormone use are associated with a higher risk for endometrial cancer [3-5]. The Type II carcinoma, which comprises mostly the serous and clear cell histological subtypes, is known to metastasize more often and to have a worse survival. In contrast to endometrial Type I carcinomas estrogen dominance does not seem to be causally linked to this type of the disease, rather higher age and previous radiation therapy of the uterus [39].

The majority of cases are classified as Type I carcinoma and comprise the endometrioid adenocarcinomas. In literature it accounts for 75-85% of all adenocarcinomas [36,37,40], which is in accordance with our study population of 72.6% endometrioid tumours.

Interestingly, we found the concomitant diagnosis of hypertension to be a negative predictor in patients diagnosed with endometrial cancer. This finding is in accordance with newly published data by Nicholas et al., who reported diabetes and hypertension to adversely affect survival and demanded to give more attention to comorbidities, since they are gaining more influence on current health care and policy [41].

Though Gd has been identified in a range of different tissue types, not all of them do indeed synthesize the protein, which is made evident by the presence and absence of Gd mRNA [14,15,42,43]. Our immunhistochemistry results were confirmed by in situ hybridization showing not only the presence of Gd in endometrial cancer but also its synthesis and thus underline its role in carcinogenesis. To our best knowledge this is the first study reporting Gd to be present on both mRNA and protein level in endometrial cancer. Moreover, existence of Gd mRNA and its close correlation to Gd protein immunoreactivity suggests that endometrial cancer cells themselves possess the ability to synthesize the Gd protein. Interestingly, no significant association of Gd mRNA and the immunosuppressive Gd glyo-epitope GdA was observed, implying that GdA positivity marks a subfraction of endometrioid cancer that cannot be predicted by sole presence of Gd mRNA. Unfortunately, due to the very limited amount of tissue available protein extraction and western blot analysis, which would allow direct quantification of Gd/GdA of the same sample, was not possible.

In hormone-dependent tumours, Gd has been described to have various effects through reduced expression of oncogens and raised expression of tumour suppressor genes. Among these Gd induced chances are reduced tumour growth, decreased metastatic properties or decreased chemoresistance [24,44]. Hautala et al. showed glycodelin to reduce breast cancer tumour growth in vivo [44]. Koistinen et al. transfected endometrial adenocarcinoma HEC-1B cells with Gd cDNA in both antisense and sense orientations [24]. They observed sense-transfected, Gd-producing carcinoma cells to have a reduced proliferation, morphologic changes, and altered expression of cancer-related genes in comparison to native and antisense-transfected carcinoma cells [24]. These results illustrate some aspects of Gd’s potential in gynecolocical cancers. In some hormone-depending tumours, Gd expression has been shown to go along with a favourable outcome, like in breast and ovarian cancer [45,46]. Results on ductal carcinoma in situ and invasive breast revealed that Gd positivity is inversely correlated with the occurrence of metastasis [45]. These data are in line with our findings, though Gd expression reached only univariate prognostice significance in Kaplan Meier analysis.

Recently histone deacetylase inhibitors (HDACIs) have been highlighted as promising new anti-cancer agents. In 2006 the HDACI suberoylanilide hydroxamic acid (SAHA, Vorinostat (rINN), Zolinza®) has been approved by the FDA for the treatment of cutaneous T-cell lymphoma and has further been evaluated in patients suffering from e.g. glioblastoma multiforme [47], non-small-cell lung [48] cancer or myelodysplastic syndroms [49]. Uchida et al. [50-52] demonstrated that SAHA is capable of up-regulating Gd in endometrial cancer and choriocarcinoma cell lines and further that SAHA induced Gd in fact influences cell differentiation and migration in the model system employed. Since we found that Gd is significantly associated with prolonged overall survival in endometrial cancer, it remains challenging to investigate whether endometrial cancer patients might also benefit from the application of SAHA. Of course randomized and properly powered clinical trials are indispensable in order to validate this hypothesis on a clinical basis.

Depending on Gd glycosylation status, it can induce apoptosis in T cells and monocytes. These in vivo results on Gd and GdA may explain the partially contradictory results in clinical studies. In contrast to Gd, we observed a poor outcome in patients expressing the immunosuppressive isoform GdA. This result was made not only on the basis of univariate but also multivariate survival analysis and is in concordance with a recently published study on ovarian cancer and GdA, where we report GdA to be a prognostic marker for poor outcome in advanced stage ovarian cancer [23]. Nevertheless, there are controversial results on Gd expression and patient survival [23,45,46,53]. These may be attributable to various mono- and polyclonal antibodies being either peptide-specific or glycosylation specific. Bearing in mind that differently glycosylated Gd isoforms may exert opposing actions may at least partially explain the conflicting research results published on this issue [54]. Functional analysis e.g. employing an endometrial cancer animal model is thus needed to further clarify the immunomodulatory actions of Gd/GdA.

## Conclusion

In conclusion, Gd and GdA are commonly expressed in endometrial cancer tissue and seem to be of relevance in tumourigenesis. They differ not only in glycosylation but also in their biological activity, since Gd is associated with a better survival, whereas GdA holds prognostic significance for a poor outcome in endometrial cancer patients. Therefore, Gd and especially GdA might help to select patients for a more individualized tumour therapy.

## Consent

As stated above the current study has been approved by the ethics committee of the Ludwig-Maximilians University Munich (approval number: 063–13) and has been carried out in compliance with the guidelines of the Helsinki Declaration of 1975. All specimens included in this study were left over samples collected during routine clinical diagnostics. Patient data were fully anonymised and the current study has been approved by the ethics committee of the LMU Munich.

## Competing interest

All authors declare to have no financial or non-financial competing interests. There is no funding source to be disclosed.

## Authors’ contributions

ML, CKK, IL made substantial contributions to conception, design and acquisition of data. SH and DM have made substantial contributions to analysis and interpretation of data. ND has been involved in drafting the manuscript and revising it critically for important intellectual content. KF and UJ have given final approval of the version to be published. In addition, KF and UJ have made substantial contributions to conception and design of the study. All authors read and approved the final manuscript.

## Pre-publication history

The pre-publication history for this paper can be accessed here:

http://www.biomedcentral.com/1471-2407/13/616/prepub

## Supplementary Material

Additional file 1 **Representative microphotographs of positive (A, B) and negative controls (C, D) for Gd (A, C) and GdA (B, D) are shown.** Placental tissue was either incubated with the respective antibodies detecting Gd (A) or GdA (B) or with the respective species matched pre-immune sera (C, D). Scale bar in A equals 100 μm and applies to A-D. Click here for file

Additional file 2 **Representative microphotographs of Gd (A, C) and GdA (B, D) in strongly (A, B; high IRS) and weakly/negatively (C, D; low IRS) stained tissue samples are shown.** Scale bar in A equals 100 μm and applies to A-D. Click here for file

Additional file 3 **Representative microphotographs of Gd (A) and GdA (B) in endometrial cancer samples of undifferentiated histology are presented.** Scale bar in A equals 100 μm and applies to A, B. Click here for file

Additional file 4 **Representative microphotographs of Gd (A, C) and GdA (B, D) in advanced (A, B; high stage) and early (C, D; low stage) staged cases are shown.** Scale bar in A equals 100 μm and applies to A-D. Click here for file

## References

[B1] Robert Koch-Institut und die Gesellschaft der epidemiologischen Krebsregister in Deutschland e.V Krebs in Deutschland 2005/2006 2010 7 Berlin: Häufigkeiten und Trends

[B2] Robert-Koch-Institut: Verbreitung von krebserkrankungen in Deutschland Entwicklung der Prävalenz zwischen 1900 und 2010 2010 Berlin

[B3] Calle EE Kaaks R Overweight, obesity and cancer: epidemiological evidence and proposed mechanisms Nat Rev Cancer 2004 13 8 579 591 10.1038/nrc1408 15286738

[B4] Wild S Pierpoint T Jacobs H McKeigue P Long-term consequences of polycystic ovary syndrome: results of a 31 year follow-up study Hum Fertil (Camb) 2000 13 2 101 105 10.1080/1464727002000198781 11844363

[B5] Beral V Bull D Reeves G Endometrial cancer and hormone-replacement therapy in the million women study Lancet 2005 13 9470 1543 1551 15866308 10.1016/S0140-6736(05)66455-0

[B6] Brinton LA Berman ML Mortel R Twiggs LB Barrett RJ Wilbanks GD Lannom L Hoover RN Reproductive, menstrual, and medical risk factors for endometrial cancer: results from a case–control study Am J Obstet Gynecol 1992 13 5 1317 1325 10.1016/S0002-9378(11)91709-8 1442985

[B7] Weiderpass E Persson I Adami HO Magnusson C Lindgren A Baron JA Body size in different periods of life, diabetes mellitus, hypertension, and risk of postmenopausal endometrial cancer (Sweden) Cancer Causes Control 2000 13 2 185 192 10.1023/A:1008946825313 10710204

[B8] Dossus L Allen N Kaaks R Bakken K Lund E Tjonneland A Olsen A Overvad K Clavel-Chapelon F Fournier A Reproductive risk factors and endometrial cancer: the European Prospective Investigation into Cancer and Nutrition Int J Cancer 2010 13 2 442 451 19924816 10.1002/ijc.25050

[B9] Mazurkiewicz JE Bank JF Joshi SG Immunocytochemical localization of a progestagen-associated endometrial protein in the human decidua J Clin Endocrinol Metab 1981 13 5 1006 1008 10.1210/jcem-52-5-1006 7014586

[B10] Joshi SG Szarowski DH Bank JF Decidua-associated antigens in the baboon Biol Reprod 1981 13 3 591 598 10.1095/biolreprod25.3.591 6796144

[B11] Morris HR Dell A Easton RL Panico M Koistinen H Koistinen R Oehninger S Patankar MS Seppala M Clark GF Gender-specific glycosylation of human glycodelin affects its contraceptive activity J Biol Chem 1996 13 50 32159 32167 10.1074/jbc.271.50.32159 8943270

[B12] Julkunen M Rutanen EM Koskimies A Ranta T Bohn H Seppala M Distribution of placental protein 14 in tissues and body fluids during pregnancy Br J Obstet Gynaecol 1985 13 11 1145 1151 10.1111/j.1471-0528.1985.tb03027.x 4063232

[B13] Koistinen H Easton RL Chiu PC Chalabi S Halttunen M Dell A Morris HR Yeung WS Seppala M Koistinen R Differences in glycosylation and sperm-egg binding inhibition of pregnancy-related glycodelin Biol Reprod 2003 13 5 1545 1551 10.1095/biolreprod.103.017830 12826581

[B14] Tse JY Chiu PC Lee KF Seppala M Koistinen H Koistinen R Yao YQ Yeung WS The synthesis and fate of glycodelin in human ovary during folliculogenesis Mol Hum Reprod 2002 13 2 142 148 10.1093/molehr/8.2.142 11818517

[B15] Chiu PC Chung MK Koistinen R Koistinen H Seppala M Ho PC Ng EH Lee KF Yeung WS Cumulus oophorus-associated glycodelin-C displaces sperm-bound glycodelin-A and -F and stimulates spermatozoa-zona pellucida binding J Biol Chem 2007 13 8 5378 5388 17192260 10.1074/jbc.M607482200

[B16] Yeung WS Lee KF Koistinen R Koistinen H Seppala M Ho PC Chiu PC Roles of glycodelin in modulating sperm function Mol Cell Endocrinol 2006 13 1–2 149 156 16413672 10.1016/j.mce.2005.12.038

[B17] Seppala M Koistinen H Koistinen R Chiu PC Yeung WS Glycosylation related actions of glycodelin: gamete, cumulus cell, immune cell and clinical associations Hum Reprod Update 2007 13 3 275 287 10.1093/humupd/dmm004 17329396

[B18] Jeschke U Toth B Scholz C Friese K Makrigiannakis A Glycoprotein and carbohydrate binding protein expression in the placenta in early pregnancy loss J Reprod Immunol 2010 13 1 99 105 10.1016/j.jri.2009.10.012 20299109

[B19] Rachmilewitz J Riely GJ Tykocinski ML Placental protein 14 functions as a direct T-cell inhibitor Cell Immunol 1999 13 1 26 33 10.1006/cimm.1998.1408 9918684

[B20] Yaniv E Borovsky Z Mishan-Eisenberg G Rachmilewitz J Placental protein 14 regulates selective B cell responses Cell Immunol 2003 13 2 156 163 10.1016/S0008-8749(03)00129-1 12826085

[B21] Rachmilewitz J Borovsky Z Riely GJ Miller R Tykocinski ML Negative regulation of T cell activation by placental protein 14 is mediated by the tyrosine phosphatase receptor CD45 J Biol Chem 2003 13 16 14059 14065 10.1074/jbc.M211716200 12556471

[B22] Mukhopadhyay D SundarRaj S Alok A Karande AA Glycodelin A, not glycodelin S, is apoptotically active. Relevance of sialic acid modification J Biol Chem 2004 13 10 8577 8584 10.1074/jbc.M306673200 14679205

[B23] Scholz C Heublein S Lenhard M Friese K Mayr D Jeschke U Glycodelin A is a prognostic marker to predict poor outcome in advanced stage ovarian cancer patients BMC Res Notes 2012 13 1 551 10.1186/1756-0500-5-551 23036050 PMC3599868

[B24] Koistinen H Seppala M Nagy B Tapper J Knuutila S Koistinen R Glycodelin reduces carcinoma-associated gene expression in endometrial adenocarcinoma cells Am J Obstet Gynecol 2005 13 6 1955 1960 10.1016/j.ajog.2005.05.073 16325596

[B25] Shabani N Kuhn C Kunze S Schulze S Mayr D Dian D Gingelmaier A Schindlbeck C Willgeroth F Sommer H Prognostic significance of oestrogen receptor alpha (ERalpha) and beta (ERbeta), progesterone receptor A (PR-A) and B (PR-B) in endometrial carcinomas Eur J Cancer 2007 13 16 2434 2444 10.1016/j.ejca.2007.08.014 17911007

[B26] Jeschke U Kuhn C Mylonas I Schulze S Friese K Mayr D Speer R Briese V Richter DU Haase M Development and characterization of monoclonal antibodies for the immunohistochemical detection of glycodelin A in decidual, endometrial and gynaecological tumour tissues Histopathology 2006 13 4 394 406 10.1111/j.1365-2559.2006.02351.x 16487361

[B27] Toth B Roth K Kunert-Keil C Scholz C Schulze S Mylonas I Friese K Jeschke U Glycodelin protein and mRNA is downregulated in human first trimester abortion and partially upregulated in mole pregnancy J Histochem Cytochem 2008 13 5 477 485 10.1369/jhc.2008.950600 18256018 PMC2324189

[B28] Mylonas I Jeschke U Kunert-Keil C Shabani N Dian D Bauerfeind I Kuhn C Kupka MS Friese K Glycodelin A is expressed differentially in normal human endometrial tissue throughout the menstrual cycle as assessed by immunohistochemistry and in situ hybridization Fertil Steril 2006 13 5 1488 1497 10.1016/j.fertnstert.2006.03.062 17070198

[B29] Bergemann C Reimer T Muller H Hosel A Briese V Friese K Jeschke U Stimulation of hCG protein and mRNA levels in trophoblast tumour cells Jeg3 and BeWo by glycodelin A Anticancer Res 2003 13 2A 1107 1113 12820356

[B30] Jeschke U Wang X Briese V Friese K Stahn R Glycodelin and amniotic fluid transferrin as inhibitors of E-selectin-mediated cell adhesion Histochem Cell Biol 2003 13 5 345 354 12743827 10.1007/s00418-003-0529-0

[B31] Jeschke U Mylonas I Kunert-Keil C Stahn R Scholz C Janni W Kuhn C Schroder E Mayr D Friese K Immunohistochemistry, glycosylation and immunosuppression of glycodelin in human ovarian cancer Histochem Cell Biol 2009 13 2 283 295 10.1007/s00418-008-0510-z 18853174

[B32] Jeschke U Bischof A Speer R Briese V Richter DU Bergemann C Mylonas I Shabani N Friese K Karsten U Development of monoclonal and polyclonal antibodies and an ELISA for the determination of glycodelin in human serum, amniotic fluid and cystic fluid of benign and malignant ovarian tumors Anticancer Res 2005 13 3A 1581 1589 16033064

[B33] Keil C Husen B Giebel J Rune G Walther R Glycodelin mRNA is expressed in the genital tract of male and female rats (Rattus norvegicus) J Mol Endocrinol 1999 13 1 57 66 10.1677/jme.0.0230057 10425447

[B34] Kunert-Keil C Jeschke U Simms G Kasper M Increased expression of glycodelin mRNA and protein in rat lungs during ovalbumin-induced allergic airway inflammation Histochem Cell Biol 2009 13 3 383 390 10.1007/s00418-008-0533-5 19002700

[B35] Remmele W Stegner HE Recommendation for uniform definition of an immunoreactive score (IRS) for immunohistochemical estrogen receptor detection (ER-ICA) in breast cancer tissue] Pathologe 1987 13 3 138 140 3303008

[B36] Bokhman JV Two pathogenetic types of endometrial carcinoma Gynecol Oncol 1983 13 1 10 17 10.1016/0090-8258(83)90111-7 6822361

[B37] Deligdisch L Holinka CF Endometrial carcinoma: two diseases? Cancer Detect Prev 1987 13 3–4 237 246 3568022

[B38] Creasman WT Endometrial cancer: incidence, prognostic factors, diagnosis, and treatment Semin Oncol 1997 13 1 S1 S140 S141-150 9045311

[B39] Kumar S Shah JP Bryant CS Seward S Ali-Fehmi R Morris RT Malone JM Jr Radiation-associated endometrial cancer Obstet Gynecol 2009 13 2 Pt 1 319 325 19155901 10.1097/AOG.0b013e3181954c5b

[B40] Denschlag D Ulrich U Emons G The diagnosis and treatment of endometrial cancer: progress and controversies Dtsch Arztebl Int 2010 13 34–35 571 577 21904591 10.3238/arztebl.2011.0571 PMC3167060

[B41] Nicholas Z Hu N Ying J Soisson P Dodson M Gaffney DK Impact of comorbid conditions on survival in endometrial cancer Am J Clin Oncol 2012 [Epub ahead of print] 10.1097/COC.0b013e318277d5f4 23241506

[B42] Julkunen M Koistinen R Sjoberg J Rutanen EM Wahlstrom T Seppala M Secretory endometrium synthesizes placental protein 14 Endocrinology 1986 13 5 1782 1786 10.1210/endo-118-5-1782 3516653

[B43] Julkunen M Koistinen R Suikkari AM Seppala M Janne OA Identification by hybridization histochemistry of human endometrial cells expressing mRNAs encoding a uterine beta-lactoglobulin homologue and insulin-like growth factor-binding protein-1 Mol Endocrinol 1990 13 5 700 707 10.1210/mend-4-5-700 1703273

[B44] Hautala LC Koistinen R Seppala M Butzow R Stenman UH Laakkonen P Koistinen H Glycodelin reduces breast cancer xenograft growth in vivo Int J Cancer 2008 13 10 2279 2284 10.1002/ijc.23773 18720404

[B45] Jeschke U Mylonas I Kunert-Keil C Dazert E Shabani N Werling M Kuhn C Janni W Gerber B Friese K Expression of glycodelin protein and mRNA in human ductal breast cancer carcinoma in situ, invasive ductal carcinomas, their lymph node and distant metastases, and ductal carcinomas with recurrence Oncol Rep 2005 13 3 413 419 15706409

[B46] Mandelin E Lassus H Seppala M Leminen A Gustafsson JA Cheng G Butzow R Koistinen R Glycodelin in ovarian serous carcinoma: association with differentiation and survival Cancer Res 2003 13 19 6258 6264 14559812

[B47] Galanis E Jaeckle KA Maurer MJ Reid JM Ames MM Hardwick JS Reilly JF Loboda A Nebozhyn M Fantin VR Phase II trial of vorinostat in recurrent glioblastoma multiforme: a north central cancer treatment group study J Clin Oncol 2009 13 12 2052 2058 10.1200/JCO.2008.19.0694 19307505 PMC2669764

[B48] Traynor AM Dubey S Eickhoff JC Kolesar JM Schell K Huie MS Groteluschen DL Marcotte SM Hallahan CM Weeks HR Vorinostat (NSC# 701852) in patients with relapsed non-small cell lung cancer: a Wisconsin Oncology Network phase II study J Thorac Oncol 2009 13 4 522 526 10.1097/JTO.0b013e3181952478 19347984 PMC3050710

[B49] Garcia-Manero G Tambaro FP Bekele NB Yang H Ravandi F Jabbour E Borthakur G Kadia TM Konopleva MY Faderl S Phase II trial of vorinostat with idarubicin and cytarabine for patients with newly diagnosed acute myelogenous leukemia or myelodysplastic syndrome J Clin Oncol 2012 13 18 2204 2210 10.1200/JCO.2011.38.3265 22585696 PMC4879705

[B50] Uchida H Maruyama T Nagashima T Asada H Yoshimura Y Histone deacetylase inhibitors induce differentiation of human endometrial adenocarcinoma cells through up-regulation of glycodelin Endocrinology 2005 13 12 5365 5373 10.1210/en.2005-0359 16123169

[B51] Uchida H Maruyama T Ono M Ohta K Kajitani T Masuda H Nagashima T Arase T Asada H Yoshimura Y Histone deacetylase inhibitors stimulate cell migration in human endometrial adenocarcinoma cells through up-regulation of glycodelin Endocrinology 2007 13 2 896 902 10.1210/en.2006-0896 17068141

[B52] Uchida H Maruyama T Ohta K Ono M Arase T Kagami M Oda H Kajitani T Asada H Yoshimura Y Histone deacetylase inhibitor-induced glycodelin enhances the initial step of implantation Hum Reprod 2007 13 10 2615 2622 10.1093/humrep/dem263 17720699

[B53] Hautala LC Greco D Koistinen R Heikkinen T Heikkila P Aittomaki K Blomqvist C Koistinen H Nevanlinna H Glycodelin expression associates with differential tumour phenotype and outcome in sporadic and familial non-BRCA1/2 breast cancer patients Breast Cancer Res Treat 2011 13 1 85 95 10.1007/s10549-010-1065-y 20676758

[B54] Dell A Morris HR Easton RL Panico M Patankar M Oehniger S Koistinen R Koistinen H Seppala M Clark GF Structural analysis of the oligosaccharides derived from glycodelin, a human glycoprotein with potent immunosuppressive and contraceptive activities J Biol Chem 1995 13 41 24116 24126 10.1074/jbc.270.41.24116 7592613

