# Supplementary figures and images for: Immunosuppressive Glycodelin A is an independent marker for poor prognosis in endometrial cancer
Source: BMC Cancer. 2013 Dec 30;13:616. doi: 10.1186/1471-2407-13-616 (PMC3898404; doi:10.1186/1471-2407-13-616)

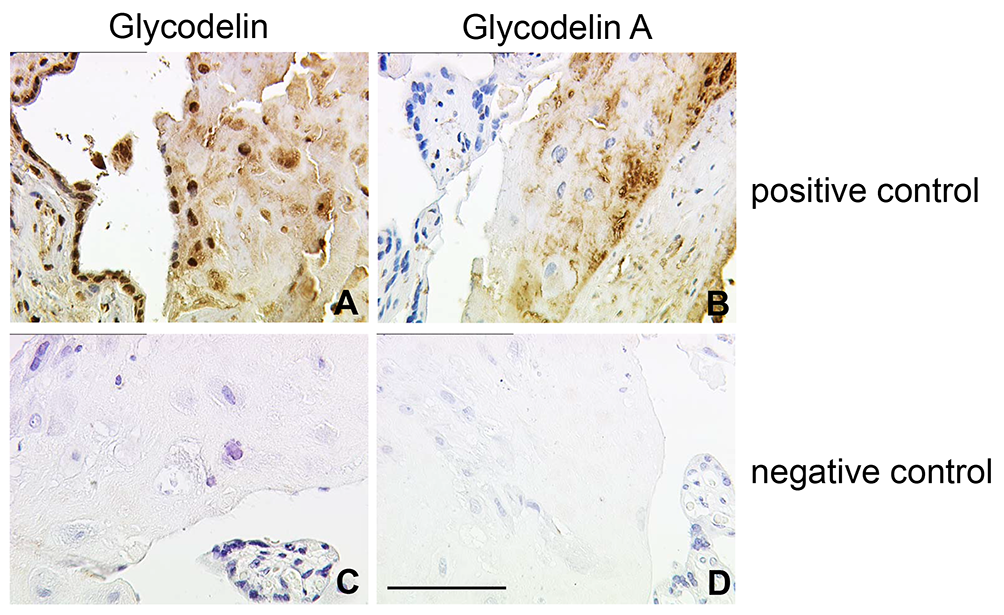

Supplement: Additional file 1 — Representative microphotographs of positive (A, B) and negative controls (C, D) for Gd (A, C) and GdA (B, D) are shown. Placental tissue was either incubated with the respective antibodies detecting Gd (A) or GdA (B) or with the respective species matched pre-immune sera (C, D). Scale bar in A equals 100 μm and applies to A-D. [file 1471-2407-13-616-S1.tiff]

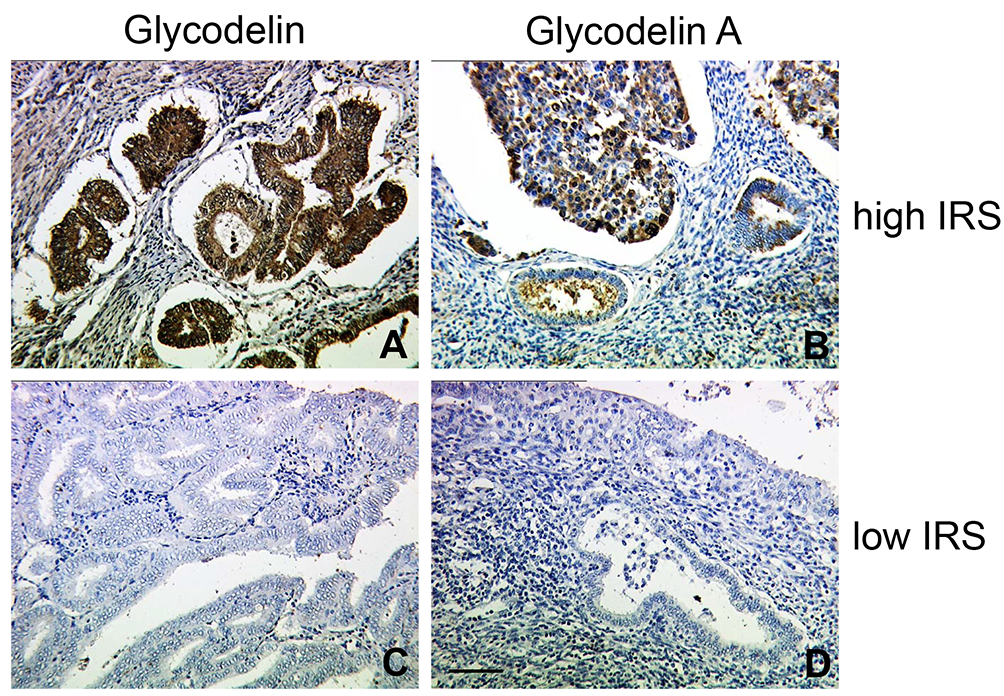

Supplement: Additional file 2 — Representative microphotographs of Gd (A, C) and GdA (B, D) in strongly (A, B; high IRS) and weakly/negatively (C, D; low IRS) stained tissue samples are shown. Scale bar in A equals 100 μm and applies to A-D. [file 1471-2407-13-616-S2.tiff]

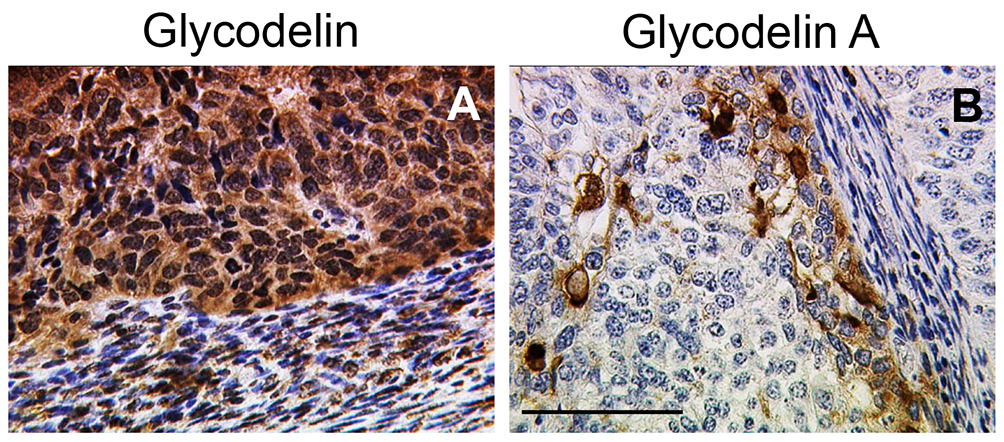

Supplement: Additional file 3 — Representative microphotographs of Gd (A) and GdA (B) in endometrial cancer samples of undifferentiated histology are presented. Scale bar in A equals 100 μm and applies to A, B. [file 1471-2407-13-616-S3.tiff]

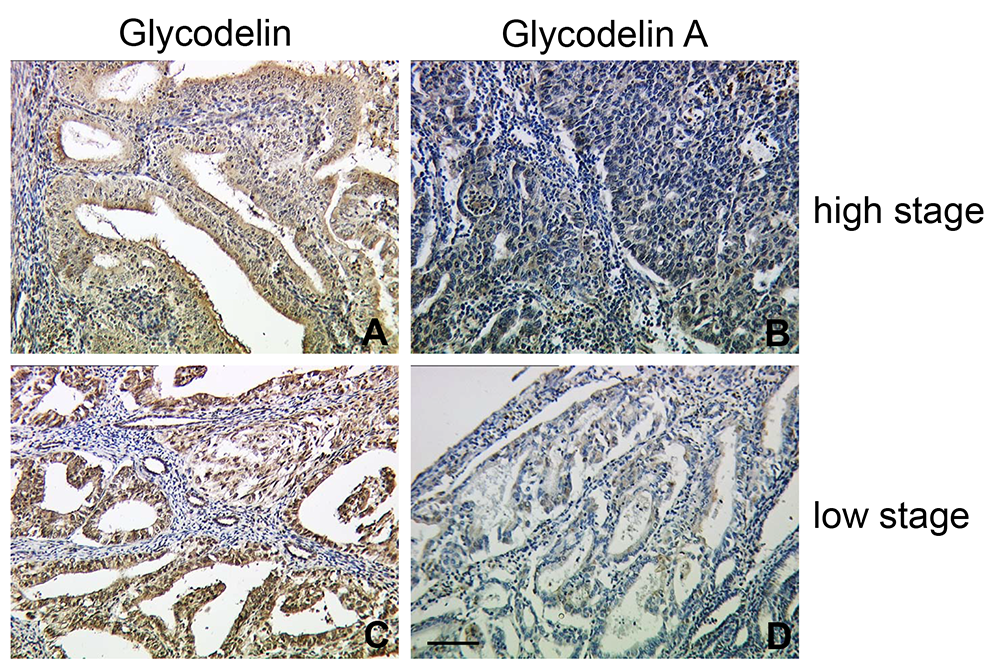

Supplement: Additional file 4 — Representative microphotographs of Gd (A, C) and GdA (B, D) in advanced (A, B; high stage) and early (C, D; low stage) staged cases are shown. Scale bar in A equals 100 μm and applies to A-D. [file 1471-2407-13-616-S4.tiff]
